# Enhancing Occupational Well-Being Among Chinese Nurses: Exploring the Mediation of Job Stress in the Relationship Between Social Support and Occupational Well-Being

**DOI:** 10.1155/jonm/2140829

**Published:** 2025-04-01

**Authors:** Yingjie Fu, Ge Qu, Jiyao Sun, Chuyun Wang, Jian Wang

**Affiliations:** ^1^Department of Social Medicine and Health Management, School of Public Health, Cheeloo College of Medicine, Shandong University, Jinan 250012, Shandong, China; ^2^NHC Key Lab of Health Economics and Policy Research, Shandong University, Jinan 250012, Shandong, China; ^3^Center for Health Management and Policy Research, Shandong University (Shandong Provincial Key New Think Tank), Jinan 250012, Shandong, China; ^4^Social Statistics, School of Social Sciences, The University of Manchester, HBS Building, Oxford Road, Manchester M13 9PL, UK; ^5^Cathie Marsh Institute for Social Research (CMI), The University of Manchester, HBS Building, Oxford Road, Manchester M13 9PL, UK; ^6^Shandong Provincial Maternal and Child Health Care Hospital Affiliated to Qingdao University, Jinan 250014, Shandong, China

**Keywords:** job stress, mediating effect, nurses, occupational well-being, social support

## Abstract

**Background:** The occupational well-being of nurses is important for nurses' human resource management and the sustainable development of hospitals. Several studies have demonstrated a positive association between social support and occupational well-being. However, the underlying mechanism behind this mechanism remains unclear. This study explored how social support influenced occupational well-being through the mediating roles of job stress.

**Methods:** This study utilized the stratified random sampling method and conducted a questionnaire survey among 450 nurses from a tertiary general hospital from July 2022 to September 2022. The surveys included the Social Support Rating Scale, Job Stress Scale, and Occupational Well-Being Scale. We also collected data on participants' sociodemographic characteristics and job-related factors. Structural equation modeling was applied to examine the associations between variables.

**Results:** The results revealed that the nurses had a moderate level of occupational well-being. Social support is positively associated with occupational well-being (*γ* = 0.600, *p* < 0.001), while job stress is negatively associated with occupational well-being (*γ* = −0.300, *p* < 0.001). Social support had a significant negative association with job stress (*γ* = −0.318, *p* < 0.001). The mediation effect shows that job stress mediated the association between social support and occupational well-being (indirect effect = 0.096, 95%CI: 0.061∼0.142), and the mediating effect of job stress can explain the 16% of the total effect of social support on occupational well-being.

**Conclusion:** This study provides evidence that the effect of social support on occupational well-being is partially mediated by job stress among nurses in China. Social support can improve nurses' occupational well-being by relieving job stress. Medical administration departments and hospital administrators should give nurses more support in their work and take targeted interventions to enhance the occupational well-being of nurses.

## 1. Introduction

As a special professional group, nurses undertake the critical responsibility of saving lives and promoting health. Their working status and ability can directly affect the lives and safety of patients [1]. The nursing staff shortage has become a critical global issue, with a negative impact on the stability of the global healthcare system and the safety of patient care [2]. Although the total number of registered nurses in China has exceeded 5 million by the end of 2023, the issue of nursing shortages remains a significant challenge for the healthcare system [3]. The lack of nursing staff allocation leads to heavy workloads and frequent overtime work. This gradually consumes their occupational well-being; consequently, their professional identity is reduced, and their turnover intention is strengthened [4–6]. Given these challenges, enhancing nurses' occupational well-being is vital for improving patient care quality, healthcare efficiency, and workforce sustainability while mitigating burnout and high turnover rates. Therefore, investigating and promoting nurses' occupational well-being has become an urgent priority for nursing management.

Occupational well-being is defined as the subjective well-being of employees in the work situation. Occupational well-being is used to measure employees' positive emotional and cognitive evaluations of work and, where possible, to expand employees' social resources, enhance organizational behavior, improve work performance, and reduce absenteeism and turnover [7]. Research showed that nurses have low or medium level of occupational well-being. A study in the United Kingdom reported that nurses had lower levels of occupational well-being [8]. Similarly, the occupational well-being of Chinese nurses is at a medium level and needs to be improved [9, 10]. For example, a study in Taiwan showed that nurses' occupational well-being was in the middle, especially in terms of psychological well-being and physical well-being [11]. The occupational well-being of nurses is not only affected by personal factors but also by work-related factors, such as professional values, career expectations, self-efficacy, leadership style, social support, workplace relationships, and job satisfaction [11–15]. Among these factors, social support plays a critical role in individual occupational well-being. This is because understanding how social support influences nurses' occupational well-being is crucial for identifying workplace stressors and support mechanisms within the healthcare setting. This knowledge could serve as a foundation for designing targeted intervention strategies to enhance nurses' occupational well-being.

Social support is defined as the variety of material and moral support that individuals receive from social relationships, including family members, friends, colleagues, partners, and associations, which play a vital role in promoting an individual's health and well-being [16]. As external resources available to individuals, social support has a certain impact on individuals' well-being outcomes. Studies have shown that there is a strong link between emotional support and occupational well-being, and that employees' stress and anxiety can be reduced through the perceived support of the organization, thereby improving occupational well-being [17, 18]. Studies have shown that the occupational well-being of nursing staff at work is closely related to the tolerance and encouragement of nursing managers and colleagues at work. The higher the support from leaders and colleagues, the higher the occupational well-being of nursing staff at work [19–21]. However, insufficient attention has been paid to how social support contributes to the enhancement of occupational well-being, and the underlying mechanism remains unclear. Therefore, this study was conducted to explore the mechanism underlying the association between social support and occupational well-being.

While social support has been identified as a crucial factor in promoting occupational well-being, particularly through emotional and organizational support, workers in the healthcare industry are more likely to experience high levels of stress than people in other occupations. For example, tense nurse–patient relationships may induce depressive symptoms and job stress in nurses [4, 22]. Unlike in developed countries, the nurse–patient relationship in China may be more challenging, potentially leading to worse occupational well-being [23]. This challenging environment, coupled with high job demands, exacerbates the stress levels nurses face in China. Job stress refers to the harmful physical and emotional reactions that occur when the demands of a job exceed the abilities, needs, or resources of the worker [24]. Increased work-related stress can damage nurses' occupational well-being, ultimately negatively affecting their caring behaviors and work productivity [25–27]. Research has demonstrated that higher work pressure is associated with lower job satisfaction, increased frustration, and a greater likelihood of job burnout, ultimately leading to a decline in nurses' occupational well-being [20, 21].

Although job stress can damage nurses' occupational well-being, according to the theoretical framework of the stress-buffering model, social support buffers an individual's response to stressors and/or stressful situations [28]. In other words, the negative effects of stress on well-being may be mitigated by social support, resulting in a positive psychological response [29]. Multiple studies have shown that social support plays an important role in coping with negative psychological aspects at work such as stress and depressive symptoms [30–32]. Therefore, job stress may serve as a mediating factor in the relationship between social support and occupational well-being. However, few studies have simultaneously examined in one study the relationship between social support, job stress and occupational well-being in nurses and their potential mechanisms.

Based on previous studies, both social support and job stress have a certain impact on occupational well-being, but the potential relationship and mechanism between the three are still unclear. Therefore, our study aims to (1) explore the level of occupational well-being of nurses, (2) explore the association between social support and occupational well-being, and (3) examine whether job stress mediated the association between social support and occupational well-being among Chinese nurses. Specifically, we proposed the following hypotheses: (1) social support would be positively associated with occupational well-being, (2) job stress would be negatively associated with social support and occupational well-being, and (3) job stress would mediate the relationship between social support and occupational well-being. The research hypotheses were examined by a mediation model.

## 2. Data and Methods

### 2.1. Study Design and Participants

Our cross-sectional study was carried out in a tertiary general hospital in Shandong Province from July 2022 to September 2022. All investigators received professional training on the questionnaire survey a few days before the formal investigation. Skilled or trained teachers and graduate students from Shandong University personally conducted structured face-to-face interviews with the survey participants in the relevant clinical departments of the hospital, with the assistance of nursing department workers. Our investigation was conducted anonymously and with the informed consent from the nurses; they were assured that participation was voluntary and that all data would remain confidential and unidentified.

First, the sample size is estimated by using the Kendall sample size estimation method. The sample size calculation was performed by the following formula: *N* = (Max[number of questionnaire items] × [5∼10]) × (1 + [10%∼30%]) (*N*: sample size; 5∼10: each questionnaire item needs at least 5 to 10 participants to ensure sample reliability and 10%∼30%: an additional percentage added to account for potential participants attrition). In our study, the maximum number of questionnaire items is 24; each item has 10 participants, and an additional percentage of 30% is added, so the required sample size is 312 at least. Then, the stratified random sampling method was used to select the participants, and it did not include practice nurses and those who worked in the hospital for less than 1 year. First, all nurses were stratified based on their hospital departments, including internal medicine, surgery, obstetrics and gynecology, emergency, and other specialized departments. Participants were then selected based on the proportional representation of nurses in each department. Ultimately, 156 participants were from internal medicine, 124 from surgery, 96 from obstetrics and gynecology, 37 from the emergency department, and 37 from other departments, resulting in a total of 450 participants. All collected questionnaires were reviewed and verified by the investigators. However, 15 questionnaires were excluded due to ineligibility. Finally, 435 participants were included in the final analysis for this study, exceeding the minimum required sample size of 312.

### 2.2. Measures

#### 2.2.1. Demographic and Work-Related Characteristics

Demographic and work-related characteristics were gender, age, marital status, number of children, educational level, sleep quality, working years, specialty, professional title, annual income, weekly working hours, and night shift per week.

#### 2.2.2. Social Support

Social support was explored using the Social Support Rating Scale (SSRS) [33]. The SSRS is a 10-item self-reported scale composed of three parts: subjective support, objective support, and support utilization. Subjective support reflects the social support that an individual feels to be understood, supported, or helped by others. Objective support refers to the practical support that an individual receives, such as financial support and practical assistance. The utilization of support reflects the extent to which social support is used, such as how individuals seek and obtain practical help in society. The total score of SSRS ranges from 12 to 66, with high scores indicating higher levels of social support. The validity and reliability of SSRS have been assessed in China, and it was considered to have good validity and reliability [34–36]. The Cronbach's *α* of this scale in the sample was 0.955.

#### 2.2.3. Occupational Well-Being

The Occupational Well-Being Scale, developed by Hu, was used to measure the occupational well-being of nurses [37]. This scale is comprised of five dimensions: physical and psychological health (six items), personal ability (six items), respectable (five items), economic income (three items), and work environment (four items). The Occupational Well-Being Scale has 24 items, with a response scale of 1 (*completely inconsistent*) to 5 (*completely consistent*), and higher scores represent better occupational well-being. The Cronbach's *α* of this scale in the sample was 0.812.

#### 2.2.4. Job Stress

Job stress was measured by the Job Stress Scale, which consists of seven items, with a response scale of 1 (*inconsistent with their own situation*) or 2 (*consistent with their own situation*) [38, 39]. In this study, the seven items included “nursing work directly affects my health.” “Nursing work creates a lot of stress.” “Even after completing nursing work, I still feel irritable and nervous.” “Health will improve if nursing work is not busy.” “Nursing work affects sleep quality.” “Major nursing tasks can be stressful.” “Nursing work interferes with other things.” The internal consistency analysis of the Job Stress Scale for our sample indicates a Cronbach's *α* of 0.846, which is consistent with values obtained in other studies ranging from 0.71 to 0.89 [39].

### 2.3. Statistical Analysis

Descriptive analysis was performed to describe the demographic characteristics of nurses, social support, job stress, and occupational well-being. A one-way analysis of variance (ANOVA) or independent *t*-test was used to compare nurses' occupational well-being by demographic and work-related characteristics. Pearson's correlation coefficient was utilized to examine the correlation between social support, job stress, and occupational well-being. Lastly, a structural equation model (SEM) was established to analyze the path relationship between social support, job stress, and occupational well-being. The steps of SEM involved in AMOS are as follows: establish the model ⟶ import relevant data ⟶ set the model parameters ⟶ evaluate the model ⟶ modify the indicators ⟶ obtain the best model. In this study, the model fitting indexes were as follows: *χ*^2^/df < 3, GFI > 0.9, AGFI > 0.9, NFI > 0.9, IFI > 0.9, CFI > 0.9, and RMSEA < 0.08. Significance testing of the mediating pathway was also performed according to methods proposed by Mackinnon [40]. Data analysis was performed using SPSS Version 24.0 (IBM Corp., Armonk, New York, United States of America) and AMOS 23.0 (IBM Corp., Armonk, New York, United States of America). All statistical analysis was two-sided, and *p* < 0.05 was established as statistically significant.

## 3. Results

### 3.1. Sample Characteristics and the Distribution of Occupational Well-Being

The study included 435 participants, and Table 1 presents the demographic and work-related characteristics of them. The average age of nurses was 32.60 ± 7.05 years old, and 95.5% were female. Most nurses were married with spouses (69.6%) and more than half had raised at least one child (63.7%). The majority of nurses held a bachelor's degree (88.2%) and worked for more than 5 years (69.9%). More than half of the nurses had poor sleep quality (59.3%). Most nurses work more than 40 h per week (66.8%) and have at least one night shift per week (71.9%). Bivariate analyses showed that the occupational well-being scores of the participants differed by sleep quality, specialty, annual income, weekly working hours, and night shift per week (*p* < 0.05).

### 3.2. Correlation Analyses of Social Support, Job Stress, and Occupational Well-Being

Table 2 presents the mean scores, standard deviations, and correlations among different social supports, job stress, and occupational well-being. The mean scores of the five dimensions of occupational well-being were 3.09 ± 1.06 (physical and psychological health), 3.73 ± 0.85 (personal ability), 4.07 ± 0.71 (respectable), 3.67 ± 0.91 (economic income), and 3.75 ± 0.89 (work environment), respectively. Social support includes subjective support, objective support, and utilization of support, with an average score of 23.89 ± 0.25, 10.03 ± 0.16, and 7.78 ± 0.09, respectively. The results indicated that social support and occupational well-being were positively correlated (*γ* = 0.356, *p* < 0.01); job stress was negatively correlated with social support (*γ* = −0.244, *p* < 0.01) and occupational well-being (*γ* = −0.161, *p* < 0.01).

### 3.3. Mediation Analyses

**Figure 1:**
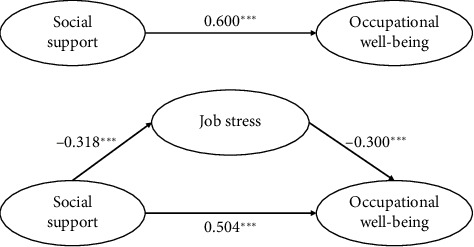
Structural equation analysis of social support, job stress, and occupational well-being.

SEM was used to quantify the hypothesized interrelationships among the three study variables. The results of SEM are presented in Tables 3, 4, and Figure 1. Regarding the quality of fit, the overall model fit indices of the valid model in Figure 1 suggested a satisfactory level with *χ*^2^/df = 2.693, GFI = 0.973, AGFI = 0.937, NFI = 0.972, IFI = 0.982. CFI = 0.982, and RMSEA = 0.062.

Results revealed a significantly positive association between social support and occupational well-being (*β* = 0.600, *p* < 0.001), which meant H1 should be accepted. Social support had a significant negative association with job stress (*β* = −0.318, *p* < 0.001), thus supporting H2. The results also indicated a negative association between job stress and occupational well-being (*β* = −0.300, *p* < 0.001), thus supporting H2.

As shown in Table 4, the indirect association between social support and occupational well-being was significant. The 95% confidence interval of mediating effects of the social support ⟶ job stress ⟶ occupational well-being pathway was (0.061, 0.142), which did not include 0, indicating that job stress mediated the relationship between social support and occupational well-being; the mediating effect of job stress can explain 16.0% of the total effect of social support on occupational well-being, thus supporting H3.

## 4. Discussion

This study is amongst the first to examine the relationship between social support and occupational well-being in Chinese nurses and the mediating role of job stress in this relationship. Results showed that Chinese nurses' occupational well-being was in the medium level; social support is positively associated with occupational well-being, while job stress is negatively associated with occupational well-being; social support is also negatively associated with job stress. This study demonstrated that job stress partially mediated the association between social support and occupational well-being, indicating that the positive influence of social support on occupational well-being seems to operate through the reduced job stress. This research contributes to the theoretical understanding of occupational well-being and offers practical policy recommendations for improving nurses' well-being and optimizing hospital human resource management.

Our study found that the nurses had a moderate level of occupational well-being, which was consistent with the results of previous studies [9, 10]. This indicates that the level of occupational well-being of nurses in China needs to be improved and should be widely concerned. The score of physical and psychological health was the lowest, indicating that the characteristics of high intensity, high pressure, and high risk of nursing work will have a great impact on the physical and psychological health of nurses. The economic income dimension had the second-lowest score. The influence of economic income on occupational well-being was also demonstrated [6, 41]. Most nurses believe that their efforts to work are not proportional to their economic income, which affects nurses' occupational well-being to a certain extent. The occupational well-being level of nurses with good sleep quality was higher than that of nurses with poor sleep quality, which was similar to the results of Yuan et al. [42]. Sleep is the most basic human need, and it helps to restore body function, regulate emotions, and consolidate memory. Lack of sleep can lead to a host of problems, such as memory loss, fatigue, emotional disorders and so on [43]. The shift and night shift system of nursing work and the special working environment may affect the quality of sleep of nurses [44]. The occupational well-being of nurses in obstetrics and gynecology was higher than that in other departments. The possible reason is that patients in obstetrics and gynecology are all female, and all of them may experience the same life experience, such as raising children, so it is easier to express emotions and communicate between nurses and patients. On the other hand, obstetrics and gynecology has its own particularity, which will welcome the arrival of new life, which will also improve the occupational well-being of nurses. Scheduling also affects nurses' occupational well-being. Nurses who worked 40 h or less per week had the highest occupational well-being. Meanwhile, nurses with less than or equal to 2 night shifts per week also had the highest occupational well-being. The majority of nurses are women who have to balance family and personal life in addition to nursing work, but this requires sufficient time and energy. We can conclude that being able to balance quality nursing services and daily life at the same time can enhance nurses' occupational well-being.

In our study, there was a positive association between social support and occupational well-being, while job stress was negatively associated with these two variables, which confirms Hypothesis 1 and Hypothesis 2. In addition, job stress was found to mediate the association between social support and occupational well-being, which confirms Hypothesis 3. The job demand–resource theory provides a theoretical basis for explaining the positive effects of social support on occupational well-being [45]. Work resources refer to the physical, psychological, social, or organizational resources that work can provide to employees, such as work autonomy, promotion and learning opportunities, harmonious colleague relations, and good working atmosphere [45]. Social support is an important psychological resource that can be provided by family, friends, colleagues, or leaders. Previous studies have made the same findings and suggested that employees' perceived social support can directly and positively affect occupational well-being [6]. Social support can also have an indirect effect on occupational well-being by alleviating job stress. A certain amount of stress can help maintain and enhance an individual's mental health and improve work productivity, but in some cases, excessive stress can lead to various psychological symptoms or disorders [46]. Rodwell's research has shown that the greater the job stress, the lower the job satisfaction, and the more likely it is to produce frustration and job burnout, which in turn leads to the decrease in nurses' occupational well-being [20]. Previous studies have shown that social support can relieve job stress [31, 32]. Nurses with more sources of social support have less perceived job stress. At the same time, individuals with more social support can cope with job stress with a positive attitude, and thus have a higher level of occupational well-being [47]. Therefore, improving nurses' social support can stimulate positive behaviors and effectively reduce job stress, thereby enhancing nurses' occupational well-being.

According to the results, giving nurses more social support is crucial. We recommend that medical administrative departments and hospital managers should create a good working atmosphere for nurses and enhance their level of social support. The perceived organizational support can be used as a protective factor to provide emotional and instrumental support for nurses to maintain their physical and mental health. Thus, on the one hand, nursing managers should offer more emotional and practical support to nurses. On the other hand, nursing managers should improve the allocation of human resources, arrange working hours reasonably, and monitor the job stress level and mental health of nurses. Meanwhile, hospital administrators should provide courses or training for nurses on how to improve their occupational well-being. Given that job stress is an important mediator, managing and reducing job stress are key factors in promoting occupational well-being. Hospital administrators should strengthen the stress management training for nurses to improve their ability to cope with job stress. Hospitals can also establish systematic staff stress management procedures. When management is informed of issues related to job stress, preventive interventions can be implemented quickly to effectively address these issues. In addition, nurses can carry out self-psychological adjustment; actively seek the support of family, colleagues, and leaders; and arrange work and life more reasonably.

Several limitations need to be considered in our study. First, the cross-sectional design does not allow us to derive causal relationships between study variables. Future research will conduct sustainable longitudinal follow-up of participants to explore the effects of dynamic changes in social support and job stress on nurses' occupational well-being. Second, the mediating role of job stress identified in this study is relatively small, which may be attributed to the influence of other unexamined factors (e.g., workload distribution, individual resilience, and coping strategies) that also contribute to the relationship between social support and occupational well-being. Future research should aim to explore these additional variables to gain a deeper understanding of the complex interplay between them. Third, the participants in this study came from a large, 2000-bed tertiary hospital, which may have a different work environment than a small hospital or clinic, thus limiting the extrapolation of this study's findings to nurses working in other types of healthcare facilities. Meanwhile, our study was only conducted in Jinan, Shandong Province, and the findings may not be applicable to other regions. Therefore, future studies will need to be explored using a more diverse sample.

## 5. Conclusion

In summary, the occupational well-being in this study was at a moderate level. Meanwhile, this study demonstrated that social support was positively associated with occupational well-being, and the effect of social support on occupational well-being was partially mediated by job stress among Chinese nurses. The results of this study provide a new perspective and thought on the relationship between social support, job stress, and occupational well-being. Medical administration departments and hospital administrators should give nurses more support in their work. Professional intervention should be targeted to help reduce the job stress and enhance the occupational well-being of nurses.

## Figures and Tables

**Table 1:** Bivariate analysis of occupational well-being with different demographic and work-related characteristics (*N* = 435).

Characteristic	*N*	%	Occupational well-being	*t*/*F*	*p* value
Gender					
Male	20	4.5	87.25 ± 14.07	0.354	0.862
Female	415	95.5	86.07 ± 10.72		
Age (years)					
≤ 29	203	46.6	86.93 ± 10.74	0.897	0.442
30∼39	162	37.2	85.61 ± 10.85		
40∼49	55	12.6	84.52 ± 11.67		
> 50	15	3.4	86.53 ± 10.19		
Marital status					
Single	132	30.3	85.80 ± 8.95	−0.412	0.681
Married	303	69.6	86.27 ± 11.64		
Number of children					
0	158	36.3	85.96 ± 9.89	0.189	0.829
1	174	40.0	86.50 ± 11.64		
2	103	23.7	85.72 ± 11.07		
Educational level					
Junior college degree	30	6.8	85.56 ± 7.98	0.054	0.948
Bachelor's degree	384	88.2	86.18 ± 11.02		
Master's degree	21	4.8	85.81 ± 12.18		
Sleep quality					
Poor	258	59.3	84.72 ± 10.97	3.273	< 0.001
Good	177	40.7	88.16 ± 10.44		
Working years					
≤ 5	131	30.1	87.66 ± 9.82	0.739	0.566
6∼10	136	31.2	86.60 ± 10.53		
11∼15	75	17.2	85.65 ± 12.00		
16∼20	51	11.7	85.18 ± 11.04		
≥ 21	42	9.6	85.42 ± 10.44		
Specialty					
Internal medicine	151	34.8	86.62 ± 9.93	3.689	0.006
Surgery	120	27.6	83.40 ± 12.44		
Obstetrics & gynecology	93	21.3	88.62 ± 10.92		
Emergency	36	8.3	84.72 ± 9.49		
Others	35	8.0	88.05 ± 8.30		
Professional title					
Primary	244	56.1	86.66 ± 10.70	0.729	0.483
Intermediate	173	39.8	85.36 ± 11.32		
Senior	18	4.1	86.38 ± 8.98		
Annual income (Chinese yuan)					
50,000∼70,000	60	13.8	86.61 ± 9.71	3.351	0.019
80,000∼100,000	141	32.5	88.29 ± 10.66		
110,000∼130,000	148	34.0	84.49 ± 10.91		
≥ 140,000	86	19.7	85.03 ± 11.48		
Weekly working hours					
≤ 40	144	33.2	87.55 ± 10.73	2.454	0.047
41∼49	226	51.9	85.78 ± 10.88		
≥ 50	65	14.9	84.13 ± 10.96		
Night shift per week					
0	122	28.1	84.78 ± 11.16	3.373	0.018
1∼2	282	64.8	88.93 ± 12.47		
≥ 3	31	7.1	87.28 ± 10.11		

**Table 2:** Correlation analysis results between key study variables (*N* = 435).

Variables	Mean (SD)	1	2	3
1. Social support	41.70 ± 0.40	1		
2. Job stress	10.80 ± 2.18	−0.244^∗∗^	1	
3. Occupational well-being	86.12 ± 10.88	0.356^∗∗^	−0.161^∗∗^	1

Abbreviation: SD, standard deviations.

⁣^∗∗^* p* < 0.01.

**Table 3:** Structural model assessment and their corresponding results.

	Standardized regression coefficient	SE	*p* value
Social support ⟶ job stress	−0.318	0.060	< 0.001
Social support ⟶ objective support	0.587	0.050	< 0.001
Social support ⟶ subjective support	0.708	0.042	< 0.001
Social support ⟶ utilization of support	0.547	0.053	
Job stress ⟶ occupational well-being	−0.300	0.045	< 0.001
Occupational well-being ⟶ physical and psychological health	0.456	0.050	< 0.001
Occupational well-being ⟶ personal ability	0.812	0.031	< 0.001
Occupational well-being ⟶ respectable	0.826	0.028	< 0.001
Occupational well-being ⟶ economic income	0.855	0.020	< 0.001
Occupational well-being ⟶ work environment	0.851	0.025	
Social support ⟶ occupational well-being	0.504	0.053	< 0.001

**Table 4:** Direct, indirect, and total effect of study variables.

Model pathways	Standardized *β* coefficient	SE	95% CI	
			Lower	Upper
Total effects				
Social support ⟶ occupational well-being	0.600	0.050	0.497	0.693
Direct effects				
Social support ⟶ job stress	−0.318	0.060	−0.433	−0.196
Job stress ⟶ occupational well-being	−0.300	0.045	−0.383	−0.205
Social support ⟶ occupational well-being	0.504	0.053	0.398	0.606
Indirect effect				
Social support ⟶ job stress⟶occupational well-being	0.096	0.020	0.061	0.142

## Data Availability

The data that support the findings of this study are available on request from the corresponding author. The data are not publicly available due to privacy or ethical restrictions.
